# Neutrophil-to-albumin ratio for predicting mortality in chronic kidney diseases: A cohort study on all-cause and cardiovascular mortality from NHANES 1999 to 2018

**DOI:** 10.1097/MD.0000000000043666

**Published:** 2025-08-08

**Authors:** Qiang Zhang, Hao Yu, He Yang, Peng-Yu Zhang, Wei-Zhe Deng, Xuan-Hua Yu

**Affiliations:** a Department of Rheumatology and Chinese Medicine, The 962nd Hospital of the PLA, Harbin, China; b The First Clinical College, Changzhi Medical College, Changzhi, China; c Department of Nephrology, The Second Affiliated Hospital of Harbin Medical University, Harbin, China; d Department of Nephrology and Rheumatology, Tongde Hospital of Zhejiang Province, Hangzhou, China; e Department of Rheumatology, People’s Hospital Affiliated to Fujian University of Traditional Chinese Medicine, Fuzhou, China.

**Keywords:** all-cause mortality, cardiovascular mortality, chronic kidney disease, neutrophil-to-albumin ratio

## Abstract

Chronic kidney disease (CKD) significantly impacts the quality of life and survival of patients globally. The neutrophil-to-albumin ratio (NAR) is a scoring system that reflects inflammation, nutritional, and mortality risk in chronic diseases. This study aims to evaluate the role of NAR in predicting CKD mortality. Data from the NHANES 1999 to 2018 were analyzed, with participants grouped by NAR quartiles. COX regression and Kaplan–Meier curves were used to examine CKD mortality. Piecewise restricted cubic spline analysis in COX regression assessed the nonlinear relationship between NAR and mortality, alongside piecewise subgroup analyses. A total of 6042 participants were included. The Q4 exhibited significantly higher all-cause mortality (24.69% vs 33.92%, *P* < .001) and cardiovascular disease (CVD) mortality (11.09% vs 14.95%, *P* = .028) compared to Q1. Kaplan–Meier curves showed Q4 had the lowest survival rate (Log-rank *P* < .001). In the final adjusted model (Model 2), Q4 had significantly higher all-cause (HR = 1.53, 95% CI = 1.35–1.74, *P* < .001) and CVD mortality (HR = 1.54, 95% CI = 1.24–1.92, *P* = .003). A nonlinear relationship was found between NAR and both all-cause (*P* < .001, *P* for nonlinear = .005) and CVD mortality (*P* < .001, *P* for nonlinear = .038), with higher risks at NAR ≥ 1.9. Our study identified a complex nonlinear relationship between NAR and CKD mortality, with NAR levels negatively correlating with survival probability, particularly in higher ranges and specific high-risk populations. These findings support the use of NAR as a tool for assessing CKD mortality risk, providing insights for early prevention, prognosis assessment, and management of CKD.

## 1. Introduction

Chronic kidney disease (CKD) is a long-term, progressive condition of kidney failure, typically characterized by a decreased glomerular filtration rate and symptoms such as proteinuria.^[1]^ CKD affects approximately 11.7% to 15.1% of the global population and is notably more prevalent in female than in male. Patients with advanced CKD often require hemodialysis, peritoneal dialysis, or kidney transplantation.^[2,3]^ CKD is one of the fastest-growing causes of death worldwide and is projected to become the fifth leading cause of death globally by 2040.^[4,5]^ The results of a large-sample retrospective study revealed that, according to the weighted data derived from a 20-year mortality cohort, the mortality rate of patients with CKD was found to be 29.17%, the mortality rate due to cardiovascular disease (CVD) was determined to be 9.79%, and deaths resulting from CVD were calculated to account for approximately 33.56% of the total number of deaths among CKD patients.^[6]^ CVD are also an independent risk factor for mortality in patients with CKD and represent a major global public health issue.^[7]^

Currently, the evaluation of poor prognosis in CKD primarily relies on serum creatinine (Scr), urinary protein, and eGFR; however, these indicators fail to fully capture the potential risk of mortality and systemic inflammation in CKD patients. Some researchers have utilized hematology inflammatory indices, such as SII, SIRI, PLR, and NLR, to evaluate the incidence and mortality risk of CKD, finding that these markers of chronic inflammation are correlated with worse disease prognosis.^[8,9]^ However, these inflammation indicators have not been incorporated into relevant nutritional parameters, and the analysis of consumption and mortality factors in chronic diseases lacks a comprehensive evaluation.

The neutrophil-to-albumin ratio (NAR) is a scoring system used to assess inflammation and nutrition in chronic diseases.^[10]^ NAR effectively reflects the systemic inflammatory state and organ function injury and is associated with the onset and poor prognosis of respiratory disease, metabolic disease, CVD, and cancers.^[11–14]^ 5083 participants who had undergone percutaneous coronary intervention were enrolled in a clinical study, through the construction of the receiver operating characteristic curve, it was demonstrated that NAR could act as a predictor for acute kidney injury (AUC = 0.64, *P* < .001).^[15]^ However, there is currently limited research on the relationship between NAR and CKD mortality, making it challenging to comprehensively assess the consistency of NAR effects on all-cause and CVD mortality in CKD.

Data from NHANES 2009 to 2018 were used to obtain a highly representative sample population through a rigorous screening process in our study. The potential association between NAR and CKD mortality was analyzed using a cohort study design. Our study aims to assist clinicians in more accurately assessing CKD mortality risk, identifying patients at high risk, and developing personalized treatment plans, thereby providing a solid foundation for implementing effective disease management strategies.

## 2. Methods

### 2.1. Study design and population

NHANES is a complex, multi-stage, nationally representative study designed to assess the health and nutritional of the civilian, noninstitutionalized population in the United States.^[16]^ It became an ongoing project with a biennial cycle. NHANES is administered by the Centers for Disease Control and Prevention (CDC) and approved by the Institutional Review Board of the National Center for Health Statistics. Written informed consent was obtained from all participants.

**Figure 1.**
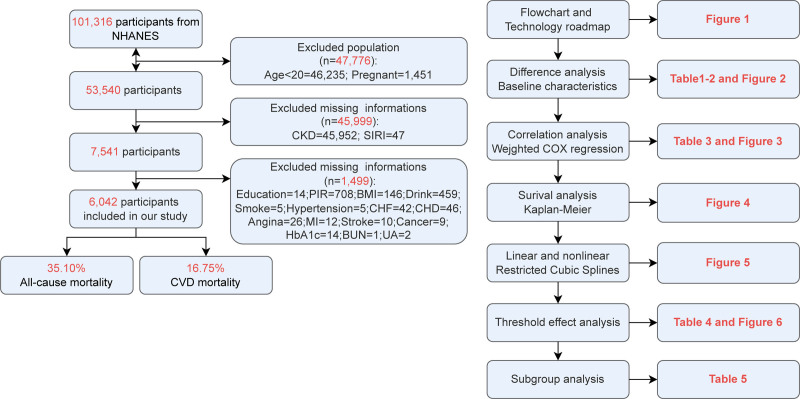
Flowcharts and technical roadmaps.

We utilized data from 10 cycles. The inclusion criteria consisted of adult participants with complete demographic, clinical, and laboratory data. 101,316 participants excluded individuals younger than 20 years or pregnant female (n = 47,776) and those missing data on key variables, including CKD, NAR, or mortality (n = 45,999). Additionally, participants missing secondary variables (education, PIR, BMI, drink, smoke, hypertension, CVD) were excluded (n = 1499). Ultimately, a total of 6042 participants were included in the analysis. The participants selection process was presented in Figure 1.

### 2.2. Defined CKD and CKD stage

In the NHANES survey, participants underwent laboratory tests and answered a series of questions to assess whether they had CKD. CKD was defined according to Kidney Disease: Improving Global Outcomes (KDIGO) criteria^[17]^: eGFR < 60 mL/min or, when eGFR ≥ 60 mL/min, urinary total protein/creatinine ratio > 30 mg/g. CKD patients were classified into stages: Stage 1 (eGFR ≥ 90 mL/min/1.73m²), Stage 2 (eGFR 60–89 mL/min/1.73m²), Stage 3 (eGFR 30–59 mL/min/1.73m²), Stage 4 (eGFR 15–29 mL/min/1.73m²), and Stage 5 (eGFR < 15 mL/min/1.73m²).

### 2.3. Defined mortality

Mortality was determined by linking with the national death index to identify mortality from any cause and from CVD as of December 31, 2019.^[18]^ Deaths were classified using the tenth edition of the International Classification of Diseases (ICD-10). CVD mortality was defined as any disease of the circulatory system as the primary cause of death (ICD-10 codes I00–I09, I11, I13, I20–I51, I60–I69).^[19]^

### 2.4. Defined neutrophil-to-albumin ratio

NAR is an indicator of inflammation and prognosis, primarily used to assess the severity of infections, inflammatory diseases, and conditions in critically patients.^[20]^ The NAR was calculated for each participant using the following formula:


$$\begin{array}{l} NAR=\frac{Neutrophil\;count\;\left(10^{3}/uL\right)}{Albumin\;\left(g/dL\right)} \end{array}$$

### 2.5. Covariates

Data on age, gender, race, education, PIR, drink, smoke, and complications were collected through household interviews using standardized questionnaires. Weight and height were measured during physical examinations at mobile medical units. Race was categorized as Mexican American, non-Hispanic White, non-Hispanic Black, or other races. Education was categorized into the following groups: less than high school, high school or equivalent, and college or higher. Drink(no/yes), smoke(no/yes), hypertension(no/yes), congestive heart failure (CHF) (no/yes), coronary heart disease (no/yes), angina (no/yes), myocardial infarction (MI) (no/yes), stroke (no/yes), and cancer (no/yes) were also recorded as covariates. Hypertension was defined as meeting one of the following criteria: doctor told you had high blood pressure, taking prescription for reducing blood pressure, taking prescribed medicine for reducing blood pressure, systolic blood pressure ≥ 135 or diastolic blood pressure ≥ 85.^[21]^ Diabetes was defined as meeting one of the following criteria: doctor told you have diabetes, taking insulin for treating diabetes now, take hypolycemic agent drugs for regulating blood glucose, fasting blood glucose ≥ 126 mg/dL, 2-hour oral glucose tolerance test blood glucose of ≥ 200 mg/dL, or glycated hemoglobin A1c (HbA1c) ≥ 6.5%.^[22]^

### 2.6. Statistical analysis

Weighted analysis of the study population over 10 cycles was performed according to the statistical methods recommended by the NHANES analysis guidelines. In the descriptive analysis, Kolmogorov-Smirnov test was used to test whether the data were normally distributed. Continuous variables with normal distributions were presented as means and standard deviations, and skewed distributions were presented as M (P25, P75). Categorical variables were expressed as frequencies. The Chi-square test was used for categorical data, and the T-test was applied to continuous variables to compare group differences. Statistical analyses and data visualizations were performed using R 4.4.2, Prism GraphPad 10.1.2, and Zstats software. *P* < .05 was considered statistically significant.

Participants were classified into 4 groups based on quartiles of NAR. Cox regression was employed to evaluate the association between NAR and CKD mortality. Differences in mortality were further illustrated through bar plots and Kaplan–Meier curves. Age, gender, race, PIR, education, BMI, drink, smoke, hypertension, diabetes, CHF, coronary heart disease, angina, MI, stroke, and cancer were included as covariates in the model.

Piecewise restricted cubic splines (RCS) were employed in Cox regression models to examine the nonlinear relationships between NAR and CKD mortality. Additionally, piecewise subgroup analyses were conducted to ascertain whether these associations held.

## 3. Result

### 3.1. Characteristic sorting

**Figure 2.**
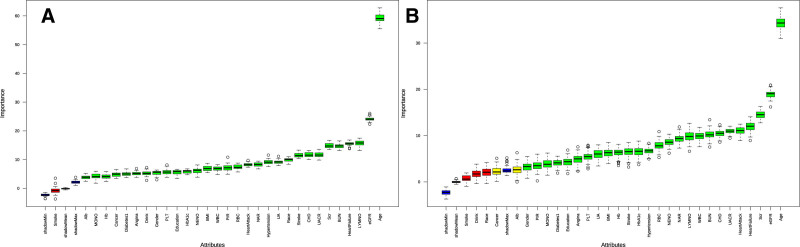
Characteristic sorting of covariates in all-cause mortality (A) and CVD mortality (B). CVD = cardiovascular disease.

Figure 2 and Figure S1, Supplemental Digital Content, https://links.lww.com/MD/P593 presented the Bourta characteristic sorting of covariates, showing NAR in all-cause (mean: 7.86, median: 7.68, normhits: 1.00) (Fig. 2A) and CVD mortality (mean: 9.39, median: 9.35, normhits: 1.00) (Fig. 2B). These results indicate that NAR as a stable importance variable in CKD.

### 3.2. Characteristics of participants

The baseline characteristics of CKD patients categorized by quartiles of NAR were presented in Tables 1 and 2. The mean age of the participants was 63 (47, 75) years, with 3163 (57.17%) female and 2879 (42.83%) male. As the NAR quartile increased, age exhibited a complex trend, initially increasing and then decreasing. BMI (28.80 vs 27.15, *P* < .001), smoking (9.50% vs 27.63%, *P* < .001), and diabetes (22.62% vs 43.13%, *P* < .001) were significantly higher in the Q1 group. Hypertension (50.37% vs 61.62%, *P* < .001), CHF (5.19% vs 11.51%, *P* < .001), and CKD stages 4 (1.72% vs 3.20%, *P* = .006) and 5 (0.89% vs 1.47%, *P* = .006) were significantly higher. Similarly, with the increase in NAR quartile, leukocyte and neutrophil (2.80 vs 4.30, *P* < .001), red blood cell, platelets (PLT), HbA1c, blood urea nitrogen, uric acid (UA), Scr, and the urinary albumin creatinine ratio were significantly elevated, while the eGFR and albumin (4.30 vs 4.20, *P* < .001) were significantly reduced.

**Table 1:** Baseline characteristics of participants with CKD.

Characteristic	NAR quantiles					*P*-value
	Overall, n = 6042	Q1, n = 1481	Q2, n = 1488	Q3, n = 1527	Q4, n = 1546	
Age	63.00 (47.00–75.00)	62.00 (46.00–74.00)	65.00 (51.00–76.00)	65.00 (48.00–75.00)	63.00 (47.00–75.00)	<.001
Gender, n%						
Male	2879 (42.83%)	674 (39.49%)	705 (42.30%)	761 (44.84%)	739 (44.38%)	.194
Female	3163 (57.17%)	807 (60.51%)	783 (57.70%)	766 (55.16%)	807 (55.62%)	
Race, n%						
Mexican American	884 (6.64%)	165 (5.12%)	218 (6.91%)	255 (7.09%)	246 (7.32%)	<.001
Non-Hispanic Black	1279 (11.47%)	518 (19.26%)	296 (10.90%)	233 (8.07%)	232 (8.31%)	
Non-Hispanic White	3044 (71.06%)	591 (65.04%)	757 (70.49%)	832 (75.80%)	864 (72.37%)	
Other race	835 (10.83%)	207 (10.58%)	217 (11.70%)	207 (9.04%)	204 (11.99%)	
Education, n%						
<High School	1938 (22.32%)	462 (21.39%)	478 (22.83%)	488 (21.05%)	510 (23.93%)	.028
>High School	2629 (51.53%)	673 (56.40%)	632 (50.84%)	662 (50.50%)	662 (48.80%)	
High School	1475 (26.15%)	346 (22.22%)	378 (26.33%)	377 (28.45%)	374 (27.27%)	
PIR	2.41 (1.29–4.42)	2.70 (1.37–5.00)	2.56 (1.37–4.48)	2.40 (1.32–4.43)	2.41 (1.29–4.42)	<.001
BMI, kg/m²	28.80 (24.90–34.00)	27.15 (23.27–31.20)	28.40 (24.85–33.00)	29.60 (25.70–35.20)	28.80 (24.90–34.00)	<.001
Drink, n%	2872 (53.82%)	724 (56.60%)	698 (53.05%)	731 (53.69%)	719 (52.17%)	.329
Smoke, n%	1017 (17.73%)	174 (9.50%)	191 (15.30%)	257 (17.51%)	395 (27.63%)	<.001
Complication, n%						
Diabetes	2362 (32.82%)	470 (22.62%)	553 (30.30%)	618 (34.09%)	721 (43.13%)	<.001
Hypertension	3783 (57.35%)	870 (50.37%)	942 (58.46%)	941 (58.33%)	1030 (61.62%)	<.001
CHF	592 (7.88%)	105 (5.19%)	141 (7.36%)	139 (7.13%)	207 (11.51%)	<.001
CHD	642 (9.40%)	130 (8.26%)	156 (8.99%)	161 (9.54%)	195 (10.67%)	.383
Angina	393 (5.93%)	78 (4.80%)	97 (6.19%)	102 (5.92%)	116 (6.69%)	.396
MI	649 (8.61%)	138 (7.95%)	137 (7.51%)	165 (8.30%)	209 (10.54%)	.077
Stroke	565 (7.59%)	132 (6.95%)	136 (7.23%)	140 (6.89%)	157 (9.19%)	.118
Cancer	1031 (17.02%)	237 (15.82%)	256 (18.35%)	273 (17.06%)	265 (16.80%)	.635
CKD stage, n%						
G1	1730 (33.45%)	491 (37.26%)	382 (30.06%)	431 (32.81%)	426 (33.80%)	.006
G2	1412 (21.73%)	320 (19.26%)	334 (20.94%)	364 (22.59%)	394 (23.84%)	
G3	2626 (41.49%)	613 (40.87%)	718 (46.64%)	661 (41.03%)	634 (37.69%)	
G4	191 (2.33%)	38 (1.72%)	40 (1.82%)	50 (2.50%)	63 (3.20%)	
G5	83 (1.00%)	19 (0.89%)	14 (0.53%)	21 (1.07%)	29 (1.47%)	

BMI = body mass index, CHD = coronary heart disease, CHF = congestive heart failure, CKD = chronic kidney disease, MI = myocardial infarction, NAR = neutrophil-to-albumin ratio, PIR = poverty income ratio.

*P* <.05 indicates statistical significance.

**Table 2:** Baseline laboratory examinations of participants with CKD.

Characteristic	Quantiles of NAR					*P*-value
	Overall, n = 6042	Q1, n = 1481	Q2, n = 1488	Q3, n = 1527	Q4, n = 1546	
WBC, 10^3^/µL	7.30 (6.00–8.60)	5.30 (4.60–5.90)	6.60 (6.00–7.20)	7.70 (7.10–8.40)	7.30 (6.00–8.60)	<.001
LYMNO, 10^3^/µL	1.90 (1.50–2.50)	1.80 (1.40–2.20)	1.90 (1.50–2.40)	2.00 (1.60–2.50)	1.90 (1.50–2.50)	<.001
MONO, 10^3^/µL	0.60 (0.50–0.70)	0.50 (0.40–0.60)	0.50 (0.40–0.70)	0.60 (0.50–0.70)	0.60 (0.50–0.70)	<.001
NENO, 10^3^/µL	4.30 (3.40–5.50)	2.80 (2.40–3.10)	3.80 (3.50–4.10)	4.80 (4.50–5.10)	4.30 (3.40–5.50)	<.001
PLT, 10^3^/µL	238.00 (199.00–284.00)	224.00 (188.00–262.00)	229.00 (195.00–271.00)	243.00 (201.00–283.00)	238.00 (199.00–284.00)	<.001
RBC, 10^6^/µL	4.59 (4.24–4.94)	4.49 (4.16–4.80)	4.58 (4.25–4.91)	4.63 (4.25–4.97)	4.59 (4.24–4.94)	<.001
Hb, %	14.00 (12.90–15.00)	13.80 (12.70–14.80)	13.90 (13.10–15.00)	14.10 (13.10–15.20)	14.00 (12.90–15.00)	<.001
HbA1c, %	5.70 (5.30–6.30)	5.60 (5.30–6.00)	5.60 (5.30–6.10)	5.70 (5.30–6.30)	5.70 (5.30–6.30)	<.001
BUN, mg/dL	16.00 (12.00–21.00)	15.00 (12.00–20.00)	16.00 (12.00–21.00)	16.00 (12.00–22.00)	16.00 (12.00–21.00)	.060
eGFR, mL/min/1.73 m²	67.95 (51.86–98.26)	72.15 (53.56–101.07)	62.14 (50.99–95.16)	67.73 (51.72–99.84)	67.95 (51.86–98.26)	.068
UACR, mg/g	41.58 (13.93–93.33)	39.30 (11.96–74.17)	37.29 (10.93–79.52)	42.30 (13.93–94.62)	41.58 (13.93–93.33)	<.001
Alb, g/dL	4.20 (4.00–4.40)	4.30 (4.10–4.50)	4.20 (4.00–4.40)	4.20 (4.00–4.40)	4.20 (4.00–4.40)	<.001
UA, mg/dL	5.80 (4.70–6.90)	5.70 (4.60–6.70)	5.80 (4.80–6.90)	6.00 (4.80–7.00)	5.80 (4.70–6.90)	.004
Scr, mg/dL	1.00 (0.78–1.23)	0.99 (0.79–1.19)	1.00 (0.80–1.24)	1.00 (0.78–1.23)	1.00 (0.78–1.23)	.360

Alb = serum albumin, BUN = blood urea nitrogen, CKD = chronic kidney disease, eGFR = estimated glomerular filtration rate, Hb = hemoglobin, HbA1c = glycated hemoglobin, LYMNO = lymphocyte, MONO = monocyte, NAR = neutrophil-to-albumin ratio, NENO = neutrophil, PLT = platelet, RBC = red blood cell, Scr = serum creatinine, UA = uric acid, UACR = urinary albumin-to-creatinine ratio, WBC = white blood cell.

*P* < .05 indicates statistical significance.

### 3.3. Cox regression analysis for quartiles of NAR and mortality in CKD

The association between NAR and CKD mortality were presented in Table 3. Both all-cause and CVD mortality rates increased progressively with higher NAR quartiles. In the unadjusted model for all-cause mortality, Q4 hazard ratio (HR = 1.55, 95% CI = 1.29–1.87, *P* < .001) showed 55% increase compared with Q1, and in Model 2, after adjusting for confounders, Q4 (HR = 1.53, 95% CI = 1.35–1.73, *P* < .001) showed 53% increase. In the unadjusted model for CVD mortality, Q4 (HR = 1.53, 95% CI = 1.21–1.92, *P* < .001) showed 53% increase in mortality compared with Q1, and in Model 2, after adjusting for confounders, Q4 (HR = 1.54, 95% CI = 1.24–1.92, *P* = .003) showed 54% increase. These results suggest a positive association between increasing NAR levels and CKD mortality.

**Table 3:** Association between NAR with all-cause and CVD mortality in CKD.

Characteristic	Quantiles of NAR				*P*/*P* for trend
	Quartile 1	Quartile 2	Quartile 3	Quartile 4	
All-cause mortstat, n%	433 (24.69)	524 (29.35)	543 (28.79)	621 (33.92)	<.001
Unadjusted	Reference	1.23 (1.00–1.51)	1.23 (1.01–1.49)*	1.55 (1.29–1.87)*	<.001
Model 1	Reference	1.10 (0.94–1.29)	1.11 (0.95–1.29)*	1.79 (1.56–2.04)*	<.001
Model 2	Reference	1.04 (0.90–1.22)	1.04 (0.88–1.22)	1.53 (1.35–1.73)*	<.001
CVD mortstat, n%	199 (11.09)	262 (15.19)	266 (13.81)	285 (14.95)	.028
Unadjusted	Reference	1.41 (1.09–1.83)*	1.32 (1.00–1.74)	1.52 (1.21–1.92)*	<.001
Model 1	Reference	1.27 (1.02–1.58)*	1.21 (0.95–1.54)	1.83 (1.46–2.28)*	<.001
Model 2	Reference	1.20 (0.97–1.50)	1.13 (0.89–1.44)	1.54 (1.24–1.92)*	.003

Model 1: adjusted for gender, age, race, education, and PIR. Model 2: adjusted for gender, age, race, education, PIR, BMI, drink, smoke, hypertension, diabetes, CHF, CHD, angina, MI, stroke, and cancer.

BMI = body mass index, CHD = coronary heart disease, CHF = congestive heart failure, CKD = chronic kidney disease, CVD = cardiovascular disease, MI = myocardial infarction, NAR = neutrophil-to-albumin ratio, PIR = poverty income ratio.

* *P*-value <.05 indicates statistical significance.

### 3.4. Kaplan–Meier analysis for quartiles of NAR and mortality in CKD

**Figure 3.**
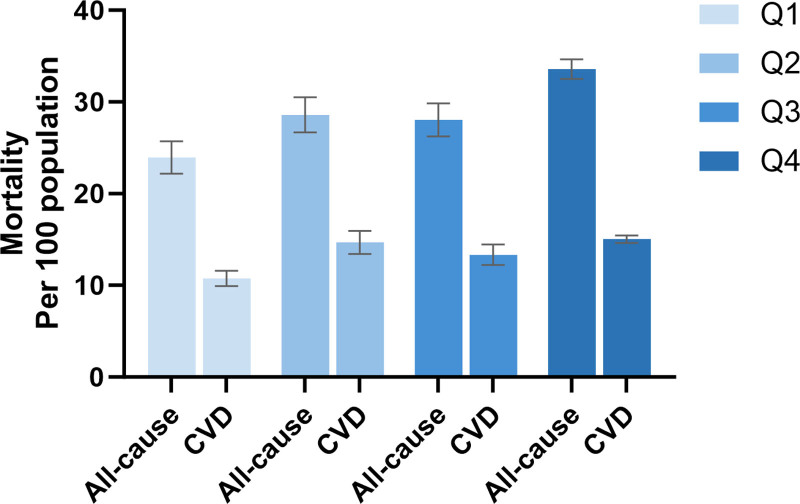
Bar graphs for quartiles of NAR in all-cause mortality and CVD mortality with CKD. CKD = chronic kidney disease, CVD = cardiovascular disease, NAR = neutrophil-to-albumin ratio.

**Figure 4.**
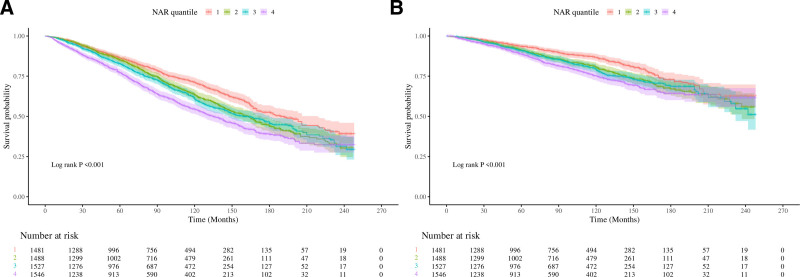
Kaplan–Meier curves for quartiles of NAR in all-cause mortality (A) and CVD mortality (B) with CKD. CKD = chronic kidney disease, CVD = cardiovascular disease, NAR = neutrophil-to-albumin ratio.

The bar graphs and Kaplan–Meier curves for all-cause (4A) and CVD (4B) mortality in CKD, grouped by quartiles of NAR were presented in Figures 3 and 4. Survival probability decreased with increasing NAR quartile values, showing a negative association between elevated NAR and reduced survival probability, with statistically significant differences between groups (Log-rank *P* < .001). Q4 exhibited the highest number of mortality and the lowest survival probability for both all-cause and CVD mortality. This trend suggests that CVD accounted for approximately half of all-cause mortality and that elevated NAR was positively associated with CKD mortality.

### 3.5. Linear and nonlinear associations between nar and mortality in CKD

**Figure 5.**
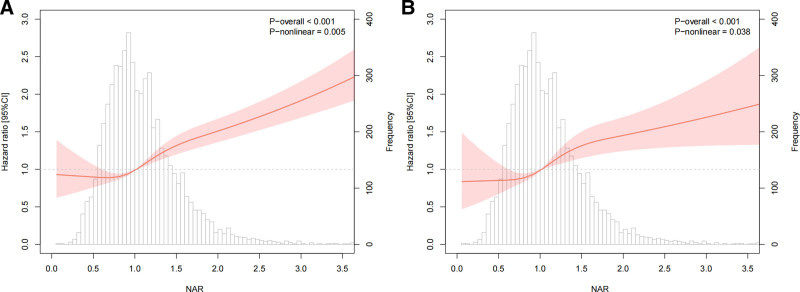
Restricted cubic spline of NAR in all-cause mortality (A) and CVD mortality (B) with CKD. CKD = chronic kidney disease, CVD = cardiovascular disease, NAR = neutrophil-to-albumin ratio.

The nonlinear associations between NAR and CKD mortality were presented in Figure 5. After adjusting the final model for all confounders, NAR was found to be associated with all-cause mortality risk in a nonlinear pattern (*P* for overall < .001, *P* for nonlinear = .005) (Fig. 5A). Above the median value (NAR = 1.02), the risk of all-cause mortality increased significantly with higher NAR. The association between NAR and CVD mortality followed a nonlinear pattern (*P* for overall <.001, *P* for nonlinear = .038) (Fig. 5B), with a significant increase in CVD mortality risk above the median value (NAR = 1.02).

### 3.6. Threshold effect analyses of NAR with mortality in CKD

**Figure 6.**
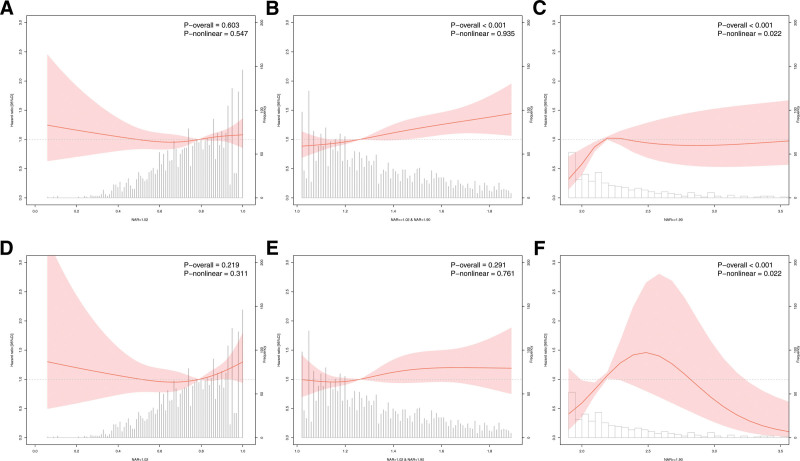
Piecewise RCS of NAR in all-cause mortality (A–C) and CVD mortality (D–F) with CKD. CKD = chronic kidney disease, CVD = cardiovascular disease, NAR = neutrophil-to-albumin ratio, RCS = restricted cubic spline.

The overall HR (HR = 1.31, 95% CI = 1.26–1.37, *P* < .001) for NAR in relation to all-cause mortality, indicating that each 1-unit increase in NAR is associated with 31.00% increase in the risk of all-cause mortality was presented in Table 4. No significant difference was observed in the threshold stratification analysis when NAR levels were below 1.02 (HR = 1.26, 95% CI = 0.92–1.72, *P* = .142). Significant differences were observed when 1.02 ≤ NAR < 1.9 (HR = 1.79, 95% CI = 1.49–2.15, *P* < .001) and when NAR ≥ 1.9 (HR = 1.23, 95% CI = 1.13–1.33, *P* < .001). In CVD mortality, the overall HR for NAR (HR = 1.28, 95% CI = 1.19–1.38, *P* < .001) indicates 28.00% increase in the risk of CVD mortality for each unit increase in NAR levels. Stratified analysis by threshold revealed no significant differences when NAR levels were below 1.02 (HR = 1.45, 95% CI = 0.93–2.28, *P* = .103) or when NAR levels were ≥ 1.9 (HR = 1.01, 95% CI = 0.72–1.42, *P* = .954). A significant difference was observed when 1.02 ≤ NAR < 1.9 (HR = 1.77, 95% CI = 1.33–2.34, *P* < .001). Model fitting was significant for both all-cause mortality risk (*P* for Log-likelihood < .001) and CVD mortality risk (*P* for Log-likelihood < .001). Figure 6 presents the linear and nonlinear associations of piecewise effects on all-cause (Fig. 6A–C) and CVD (Fig. 6D–F) mortality.

**Table 4:** Threshold efect analyses of NAR with all-cause and CVD mortality in CKD.

Characteristic	HR (95% CI)*	*P*-value
All-cause mortality		
Total	1.31 (1.26–1.37)	<.001
Three-piecewise Cox regression model		
NAR < 1.02	1.26 (0.92–1.72)	.142
1.02 ≤ NAR < 1.9	1.79 (1.49–2.15)	<.001
NAR ≥ 1.9	1.23 (1.13–1.33)	<.001
Log-likelihood ratio	–	<.001
CVD mortality		
Total	1.28 (1.19–1.38)	<.001
Three-piecewise Cox regression model		
NAR < 1.02	1.45 (0.93–2.28)	.103
1.02 ≤ NAR < 1.9	1.77 (1.33–2.34)	<.001
NAR ≥ 1.9	1.01 (0.72–1.42)	.954
Log-likelihood ratio	–	.006

CI = confidence interval, CKD = chronic kidney disease, CVD = cardiovascular disease, HR = hazard ratio, NAR = neutrophil-to-albumin ratio.

* *P*-value <.05 indicates statistical significance.

### 3.7. Three-piecewise subgroup analyses of NAR with mortality in CKD

The results of subgroup stratified analyses examining the association between NAR with all-cause and CVD mortality were presented in Table 5. When NAR exceeded 1.90, males (HR = 1.80, 95% CI = 1.26–2.56) and middle-aged and young adults exhibited the highest all-cause mortality (HR = 2.93, 95% CI = 1.74–4.93), while CKD Stage 5 (HR = 3.95, 95% CI = 0.83–18.79) had the highest mortality risk. No significant interaction effect was observed among any of the subgroups (*P* for interaction > .05). When NAR exceeded 1.90, female (HR = 2.12, 95% CI = 1.37–3.27), middle-aged and young adults (HR = 3.31, 95% CI = 1.47–7.45), and CKD Stage 5 (HR = 5.59, 95% CI = 1.14–27.30) were associated with the highest risk of mortality. Subgroup analysis results revealed a weak interaction between NAR and CKD stage in relation to CVD mortality (*P* for interaction = .048), while robust results were observed in the remaining subgroups (*P* for interaction > .05).

**Table 5:** Three-piecewise subgroup analysis of NAR with all-cause and CVD mortality in CKD.

Subgroup covariates	All-cause mortality HR (95% CI)			*P* for interaction	CVD mortality HR (95% CI)			*P* for interaction
	NAR < 1.02	1.02–1.9	NAR ≥ 1.9		NAR < 1.02	1.02–1.9	NAR ≥ 1.9	
Overall	0.84 (0.74–0.96)	1	1.62 (1.28–2.06)	**–**	0.91 (0.76–1.09)	1	1.81 (1.30–2.53)	**–**
Gender								
Male	0.84 (0.70–1.00)	1	1.80 (1.26–2.56)	.640	0.88 (0.69–1.13)	1	1.54 (0.94–2.50)	.369
Female	0.86 (0.70–1.06)	1	1.56 (1.12–2.19)		0.95 (0.74–1.21)	1	2.12 (1.37–3.27)	
Age								
< 60	1.09 (0.74–1.61)	1	2.93 (1.74–4.93)	.113	1.30 (0.68–2.47)	1	3.31 (1.47–7.45)	.066
≥ 60	0.72 (0.63–0.81)	1	1.48 (1.15–1.90)		0.77 (0.64–0.91)	1	1.70 (1.20–2.41)	
CKD stage								
G1	0.91 (0.66–1.26)	1	2.19 (1.25–3.82)	.133	1.09 (0.66–1.81)	1	1.63 (0.62–4.29)	.048
G2	1.00 (0.78–1.28)	1	1.55 (0.99–2.43)		1.10 (0.76–1.58)	1	1.28 (0.61–2.68)	
G3	0.74 (0.62–0.87)	1	1.55 (1.10–2.19)		0.75 (0.59–0.94)	1	2.23 (1.53–3.27)	
G4	0.86 (0.51–1.46)	1	1.41 (0.51–3.88)		1.28 (0.59–2.80)	1	1.23 (0.27–5.56)	
G5	2.06 (0.70–6.06)	1	3.95 (0.83–18.79)		2.10 (0.51–8.64)	1	5.59 (1.14–27.30)	

CI = confidence interval, CKD = chronic kidney disease, CVD = cardiovascular disease, HR = hazard ratio, NAR = neutrophil-to-albumin ratio.

*P*-value < .05 indicates statistical significance.

## 4. Discussion

Data from NHANES 1999 to 2018, analyzed 6042 adult participants to investigate the relationship between the NAR and CKD mortality, with a focus on assessing the predictive value of NAR for CKD mortality in our study. The robustness of the association between NAR and CKD mortality was confirmed through several analytical approaches, including weighted quartile group variance analysis, survival analysis, Cox regression models, segmented RCS analysis, and subgroup analysis. Our findings indicate a complex, positive, and nonlinear relationship between NAR levels and CKD mortality. These results imply that controlling NAR may serve as an effective strategy to reduce mortality risk in CKD patients.

Kidney function is impaired in patients with CKD, leading to the accumulation of metabolic waste and toxins in the body. This results in inflammation and immune dysfunction, which are key risk factors for the progression of CKD.^[23]^ Chronic low-grade inflammation has been increasingly recognized as a hallmark of CKD, contributing to heightened disease activity, increased CVD events, and elevated mortality.^[24]^ Neutrophils, as crucial inflammatory cells with immune functions, release a range of pro-inflammatory mediators, including IL-1, TNF-α, IL-6, and reactive oxygen species, which can exacerbate tissue damage.^[25,26]^ These inflammatory factors are primarily derived from the bloodstream and are widely distributed across nearly all tissues and organs. The kidneys, which receive 25% of the body’s total blood volume, are particularly vulnerable to inflammatory attacks due to the absence of antioxidant and anti-inflammatory defense mechanisms, unlike those found in the liver.^[27]^ In addition to their role in waste excretion, the kidneys also function as metabolic organs. The nutritional of CKD patients is often compromised due to disturbances in protein, fat, mineral, and vitamin metabolism.^[28]^ Protein-energy wasting syndrome, a common complication of CKD, manifests as weight loss, muscle wasting, and decreased serum albumin levels, primarily driven by inadequate nutrient intake, metabolic changes associated with chronic disease, and increased protein catabolism.^[29,30]^ Furthermore, impaired kidney function results in excessive protein breakdown and excretion, leading to a negative nitrogen balance that exacerbates malnutrition.^[31]^ The interplay of inflammation, immune dysfunction, and nutritional depletion accelerates CKD progression and increases mortality risk.

Participants with high NAR levels exhibited higher Scr values, lower eGFR, and significantly elevated urinary albumin creatinine ratio and UA compared to those with low NAR levels. As a composite index, NAR effectively reflects the systemic level of inflammation, with an increase in neutrophil count being a primary contributor to the elevated NAR values.^[32]^ In patients with CKD, both cellular and humoral immunodeficiencies coexist with the activation of immune cells, leading to an increase in neutrophils and a reduction in lymphoid-associated cells, which are common hematological findings in CKD patients.^[33–35]^ A study conducted at Sichuan University, China, involving 966 CKD patients, found that neutrophil levels were significantly higher in individuals at high risk for progression to ESRD (5.45 [4.38–6.87] vs 3.71 [2.93–4.48], *P* < .001).^[36]^

All-cause and CVD mortality were significantly higher in CKD participants with high NAR compared to those with low NAR. A study conducted by Xiangya Hospital, Central South University, analyzed data from NHANES 2003 to 2010 involving 11,262 participants, of which 3015 (26.77%) had CKD. This study found that inflammatory markers, including monocyte-to-lymphocyte ratio, neutrophil-to-lymphocyte ratio (NLR), and C-reactive protein (CRP), were positively correlated with the incidence of CKD. Notably, NLR (0.60; 95% CI = 0.59–0.63) demonstrated good stability in predicting mortality, with a sensitivity of 53.58% and specificity of 65.80%.^[37]^ Similarly, a Romanian study of 461 CKD patients reported that those in the high NLR group (40.12% vs 1.97%, *P* < .001) had significantly higher 30-day mortality, prolonged hospital stays, and an increased number of dialysis sessions.^[38]^ Although the exact populations, variables, and outcomes differ, the results of these studies align with those of the present study.

Gender, age, CVD, and other factors significantly influence both total and CVD mortality in CKD. In our subgroup analysis, participants with high NAR exhibited a higher risk of all-cause mortality in male and a higher risk of CVD mortality in female. Globally, CKD prevalence is generally lower in men than in female, yet men tend to have higher blood pressure levels and greater metabolic burdens.^[39,40]^ Female, on the other hand, often experience a longer disease trajectory, which is associated with estrogenic imbalances and a heightened immune-response stress state.^[41]^ Research has shown an increase in the prevalence of MI in female aged 35 to 54 years, while a corresponding decrease is observed in age-matched men.^[42]^ CVD is a common comorbidity in CKD and a leading cause of mortality in these patients. The interaction between CKD and CVD is complex, with CVD significantly exacerbating the disease burden of CKD. Both conditions share numerous risk factors and demonstrate a pronounced “kidney-heart” axis effect.^[43–45]^

We observed an increase in both all-cause and CVD mortality among individuals under 60 years of age with high NAR group in our subgroup analysis. The Kaplan–Meier curve indicated a more rapid decline after 10 years of disease progression, suggesting a higher mortality rate at this stage. Kidney function may deteriorate more quickly in younger patients over a shorter duration, and younger individuals are at greater risk of secondary kidney diseases (diabetes, systemic lupus erythematosus, drug-induced kidney injury), which accelerate the progression of renal failure.^[46]^ Although the absolute risk of mortality tends to be higher in older patients due to limited lifespan, a Belgian study stratified by age revealed that, in the 18 to 54 year age group, the risk of mortality increased when eGFR fell below 75 mL/min/1.73 m². In the 55 to 64 year age group, this increase occurred when eGFR fell below 60 mL/min/1.73 m², while for individuals over 65, the risk only became significant when eGFR dropped below 45 mL/min/1.73 m².^[47]^ A Swedish study involving 7388 patients with CKD-G3, 18,282 with CKD-G4, and 9410 with CKD-G5 found that 19.6% (95% CI = 19.2–20.0) of patients progressed, and 10.1% (95% CI = 9.9–10.3) died. Female had a lower risk of CKD progression (HR = 0.88, 95% CI = 0.85–0.92), as well as a lower risk of all-cause (HR = 0.90, 95% CI = 0.85–0.94) and CVD mortality (HR = 0.83, 95% CI = 0.76–0.90) compared to men.^[48]^ The discrepancies between our study and previous research underscore the importance of stratifying CKD studies by inflammatory markers, gender, and age.

Elevated neutrophils and decreased albumin are one of the clinical manifestations of immune, inflammatory and nutritional metabolic disorders, and they are also the main driving factors for the increase in NAR that we have observed. Controlling potential infection risks and regulating the use of immunomodulatory drugs requires a careful assessment of risks and benefits.^[49,50]^ According to the KDIGO guidelines, it is recommended that the daily protein intake for CKD patients be set at 0.6 to 0.8 g/kg. For CKD patients who exhibit unstable metabolism, the recommendation is that low-protein or very low-protein diets not be adopted. For patients suffering from severe hypoalbuminemia, the nutritional status can be rapidly corrected and essential amino acids can be supplemented through short-term intravenous infusion of albumin or high-energy nutritional agents, thereby maintaining a positive nitrogen balance.^[1]^ The data on the all-cause mortality and cardiovascular mortality factors of the NAR segments collected by us provide data support for the risk stratification process in clinical practice.

The strength of our study lies in its large-sample size and rigorous methodological design. Based on nationally representative data from the United States, our analysis is highly generalizable. Moreover, the study adhered strictly to the STROBE guidelines, incorporating adequate variable adjustment, multiple models, and subgroup analyses, along with longitudinal mortality data, to strengthen causal inferences and ensure the robustness and reliability of the findings. However, it is subject to certain limitations, including the absence of periodic laboratory measurements. Additionally, NAR, as an inflammation marker, may be influenced by other unidentified factors. Future longitudinal clinical studies and randomized controlled trials are needed to further explore the relationship between NAR and both all-cause and CVD mortality, providing stronger evidence for the use of NAR in predicting mortality risk in CKD.

## 5. Conclusion

A complex positive nonlinear relationship was observed between NAR and CKD mortality in our study. NAR levels were negatively correlated with survival probability, particularly in higher NAR ranges and specific high-risk populations. As a potential tool for assessing mortality risk in CKD, NAR demonstrates promising clinical applications. The findings offer new insights and a foundation for the early prevention, prognosis assessment, and survival management of CKD.

## Acknowledgments

Our research group is grateful to the Fujian Provincial Health Commission support for our study.

## Author contributions

**Conceptualization:** Qiang Zhang.

**Data curation:** Hao Yu, Peng-Yu Zhang.

**Formal analysis:** Qiang Zhang.

**Funding acquisition:** Qiang Zhang.

**Investigation:** Hao Yu, Peng-Yu Zhang.

**Methodology:** He Yang.

**Project administration:** Qiang Zhang.

**Resources:** Qiang Zhang.

**Software:** Qiang Zhang.

**Supervision:** He Yang.

**Validation:** Qiang Zhang.

**Visualization:** Qiang Zhang.

**Writing – original draft:** Qiang Zhang.

**Writing – review & editing:** Wei-Zhe Deng, Xuan-Hua Yu.

## Supplementary Material


