# Supplementary material for: Neutrophil-to-albumin ratio for predicting mortality in chronic kidney diseases: A cohort study on all-cause and cardiovascular mortality from NHANES 1999 to 2018
Source: Medicine (Baltimore). 2025 Aug 8;104(32):e43666. doi: 10.1097/MD.0000000000043666 (PMC12348407; doi:10.1097/MD.0000000000043666)
Supplement: Supplementary file 1 [file medi-104-e43666-s001.pdf]

```
             meanImp  medianImp     minImp     maxImp normHits  decision
Age         63.1254587 62.8137405 59.5733813 67.7066435  1.00000 Confirmed
Gender       5.2992250  5.2585300  3.5607445  7.1025424  1.00000 Confirmed
Race         9.4842525  9.6362509  7.4978644 11.2778638  1.00000 Confirmed
Education    4.8665415  4.9476972  2.8451497  6.8231332  1.00000 Confirmed
PIR          7.6820531  7.6463673  5.7012417  9.6221305  1.00000 Confirmed
BMI          6.7472005  6.6337371  4.8517420  8.6069504  1.00000 Confirmed
Drink        4.0835013  4.0109188  2.7736251  5.7937656  0.96875 Confirmed
Smoke       -0.2360662 -0.1617599 -1.4064372  0.8572572  0.00000  Rejected
Hypertension 9.3513915  9.3642143  6.9721910 10.4756635  1.00000 Confirmed
Diabetes     4.7991616  4.9140811  2.7479619  6.3521207  0.93750 Confirmed
CHF         12.0048137 12.0343619 10.7100581 13.4030510  1.00000 Confirmed
CHD          9.0309690  8.9574474  7.9190802 10.6237223  1.00000 Confirmed
Angina       3.3534774  3.3880152 -0.1552828  5.4401313  0.81250 Confirmed
MI           6.2478354  6.1968686  4.7672421  7.7782866  1.00000 Confirmed
Stroke       8.5302336  8.6564994  6.5337790  9.5186980  1.00000 Confirmed
CVD         16.2572976 16.2960123 14.5878768 17.8585258  1.00000 Confirmed
WBC          6.4013511  6.4975139  4.4156653  8.2229372  1.00000 Confirmed
LYMNO       15.2735371 15.3885242 12.8831544 17.5705400  1.00000 Confirmed
MONO         4.2541158  4.1436751  2.7189988  6.0210078  0.93750 Confirmed
NENO         6.4444616  6.1250813  5.1717971  9.2135002  1.00000 Confirmed
PLT          5.5910724  5.5296688  3.4762089  7.6972941  0.96875 Confirmed
RBC          6.5343560  6.5423123  5.0417270  8.3742380  1.00000 Confirmed
Hb           3.8636990  3.8271867  1.9757246  5.3672529  0.87500 Confirmed
HbA1c        6.2054061  6.2668211  4.1880205  8.0929995  1.00000 Confirmed
eGFR        23.5015020 23.6311971 21.7033707 25.8059908  1.00000 Confirmed
UACR        12.3431431 12.4752136 10.9211570 13.4389431  1.00000 Confirmed
CKD_stage   13.3420932 13.3116753 12.1059954 14.6441899  1.00000 Confirmed
Cancer       3.9370106  3.8569847  1.1206808  5.8421218  0.93750 Confirmed
Alb          4.2010214  4.1495015  2.4248175  6.1848100  0.93750 Confirmed
BUN         13.0832365 13.1284302 11.5993405 14.5465163  1.00000 Confirmed
UA          10.2913390 10.3553628  7.9522221 12.3644401  1.00000 Confirmed
Scr         14.7793047 14.8088614 13.4630602 16.3241577  1.00000 Confirmed
NAR          7.8632330  7.6803071  6.2974710 10.6284872  1.00000 Confirmed
y ~ Age + Gender + Race + Education + PIR + BMI + Drink + Hypertension +
    Diabetes + CHF + CHD + Angina + MI + Stroke + CVD + WBC +
    LYMNO + MONO + NENO + PLT + RBC + Hb + HbA1c + eGFR + UACR +
    CKD_stage + Cancer + Alb + BUN + UA + Scr + NAR
<environment: 0x000001dcdd117850>
```

```
               meanImp  medianImp     minImp    maxImp   normHits  decision
Age          34.4538501 34.3337604 31.5347189 37.199640 1.00000000 Confirmed
Gender        3.0956410  3.1313956  0.8500922  4.954681 0.79797980 Confirmed
Race          1.4177818  1.2918805 -0.5825454  3.510582 0.13131313  Rejected
Education     3.8588974  3.9171135  1.3201525  6.335372 0.91919192 Confirmed
PIR           3.5876623  3.6288827  1.3339601  6.284332 0.88888889 Confirmed
BMI           6.0921362  6.1868947  3.7422142  8.430728 1.00000000 Confirmed
Drink         1.4374897  1.5081846 -1.2658614  3.956022 0.13131313  Rejected
Smoke         0.7234646  0.4288208 -0.5076109  2.323801 0.01010101  Rejected
Hypertension  6.7264958  6.7635917  4.6950690  9.073942 1.00000000 Confirmed
Diabetes      3.8587755  3.8280045  1.8207745  6.045758 0.92929293 Confirmed
CHF           8.6534885  8.6855281  7.0399942 10.316299 1.00000000 Confirmed
CHD           7.9005788  7.8779448  5.7597472  9.511719 1.00000000 Confirmed
Angina        3.4180203  3.3906974  1.5654635  5.838439 0.86868687 Confirmed
MI            8.2121781  8.2483830  6.3291597  9.967583 1.00000000 Confirmed
Stroke        5.7057630  5.7442105  3.9180141  8.070640 1.00000000 Confirmed
CVD          13.2647996 13.2303264 11.8249342 15.430386 1.00000000 Confirmed
WBC           9.8669044  9.9179368  8.0328664 12.136712 1.00000000 Confirmed
LYMNO         9.5758797  9.4629903  7.3927611 12.552431 1.00000000 Confirmed
MONO          3.7545005  3.7139313  1.6360329  6.504011 0.90909091 Confirmed
NENO          8.5628624  8.5038752  6.4480573 11.007955 1.00000000 Confirmed
PLT           5.6222259  5.6642333  3.2561536  8.306746 1.00000000 Confirmed
RBC           7.1157464  7.1762980  4.6736650  9.156325 1.00000000 Confirmed
Hb            5.8813401  5.8274292  3.5919986  8.563284 1.00000000 Confirmed
HbA1c         6.5397784  6.4372225  4.1534726  9.974220 1.00000000 Confirmed
eGFR         18.4350269 18.4563692 16.2631539 19.997446 1.00000000 Confirmed
UACR         11.5822154 11.5605844  9.9818266 12.917268 1.00000000 Confirmed
CKD_stage    10.7109649 10.6410550  8.9116970 12.297925 1.00000000 Confirmed
Cancer        2.1714897  2.2298531 -0.2544809  4.705607 0.45454545 Tentative
Alb           2.5658929  2.5889409  0.1632066  5.243204 0.66666667 Confirmed
BUN           8.9435327  8.8900643  6.9490808 11.644577 1.00000000 Confirmed
UA            6.4557774  6.5167094  3.5582293  9.037448 1.00000000 Confirmed
Scr          15.0931498 15.1109342 12.9782386 17.352383 1.00000000 Confirmed
NAR           9.3883639  9.3552378  7.3895459 11.955352 1.00000000 Confirmed
y ~ Age + Gender + Education + PIR + BMI + Hypertension + Diabetes +
    CHF + CHD + Angina + MI + Stroke + CVD + WBC + LYMNO + MONO +
    NENO + PLT + RBC + Hb + HbA1c + eGFR + UACR + CKD_stage +
    Cancer + Alb + BUN + UA + Scr + NAR
<environment: 0x000001dcdc51a900>
```
